# Visual search and the N2pc in children

**DOI:** 10.3758/s13414-015-0833-5

**Published:** 2015-02-14

**Authors:** Jane W. Couperus, Colin Quirk

**Affiliations:** Adele Simmons Hall, Cognitive Sciences, Hampshire College, Amherst, MA 01002 USA

**Keywords:** Visual selective attention, N2pc, Visual search, Children, Event-related potentials

## Abstract

While there is growing understanding of visual selective attention in children, some aspects such as selection in the presence of distractors are not well understood. Adult studies suggest that when presented with a visual search task, an enhanced negativity is seen beginning around 200 ms (the N2pc) that reflects selection of a target item among distractors. However, it is not known if similar selective attention-related activity is seen in children during visual search. This study was designed to investigate the presence of the N2pc in children. Nineteen children (ages 9–12 years) and 21 adults (ages 18–22 years) completed a visual search task in which they were asked to attend to a fixation surrounded by both a target and a distractor stimulus. Three types of displays were analyzed at parietal electrodes P7 and P8; lateral target/lateral distractor, lateral target/midline distractor, and midline target/lateral distractor. Both adults and children showed a significant increased negativity contralateral compared to ipsilateral to the target (reflected in the N2pc) in both displays with a lateral target while no such effect was seen in displays with a midline target. This suggests that children also utilized additional resources to select a target item when distractors are present. These findings demonstrate that the N2pc can be used as a marker of attentional object selection in children.

Searching through a visual scene requires a number of complex processes, such as selective attention, which must be utilized to enhance processing of the attended items while simultaneously irrelevant items must be ignored. The neurological correlates of these processes have been under study for over two decades (Luck & Hillyard, 1994a, b; Taylor & Khan, 2000). However, despite these studies, numerous questions remain. Among them, how might selective attention in visual search in children differ from that of adults and do children use top-down guided attentional processes to select stimuli and suppress distractors during visual search?

Behavioral studies of visual search in children suggest that there may be differences between adults and children. In addition to adults having faster search times than children (Miller, 1978), children show both differences (Donnelly et al., 2007; Miller, 1978) and similarities (Gerhardstein & Rovee-Collier, 2002; Trick & Enns, 1998) in *how* they search. Some evidence suggests that sensory-motor maturation as well as development of cognitive abilities contribute to differences in performance on visual search tasks (Grubert, Indino, & Krummenacher, 2014). Moreover, there is some evidence that the mechanisms supporting selective attention in visual search may differ between adults and children. For example, a recent study by Couperus, Hunt, Nelson, and Thomas (2011) used a contextual cueing paradigm to examine selective attention and implicit learning in 10-year-old children. The contextual cueing paradigm consists of a visual search task in which some or all of the items within the visual search repeat the arrangement of locations in relation to the target over time. As the locations of the items are learned, search becomes faster. However, this paradigm can also be used to look at selective attention by including two colors of items through which to search (here green and red items) and varying which color(s) are attended and which contain relevant information. By including displays that contain repeating location information in both color items or instead in either the attended or unattended color alone, it is possible to show that learning only occurs if attended items contain the repeating location information. However, children, unlike adults, only show learning if all of the items (i.e., both colors) contain repeating location information or if there are few non-repeating items in the unattended color. Intriguingly, if the number of total items remains constant and the ratio of attended to unattended items is varied, search times increase with the number of attended items to be searched at the same rate for both adults and children. However, learning is impacted by the number of unattended items for children while it makes no difference for adults. In other words, the attended items are appropriately selected for during the search, but the unattended items are not sufficiently filtered.

This discrepancy between selection and filtering in children (Couperus et al., 2011) along with other studies suggesting differences in how children perform visual search (Donnelly et al., 2007; Miller, 1978) suggests that there may also be differences between adults and children in the underlying neurological processes that support selective attention in visual search. Specifically, there may be differences in the ability to use an attentional set to guide attention and in turn the implementation of that attentional set during stimulus processing when completing a visual search. However, previous visual search studies in children have either only used behavioral methods or have focused on components that while reflecting selection during stimulus processing may not reflect other attentional processes involved in visual search.

Selective attention, specifically visual selective attention, has traditionally been described through a sensory gain model (Hillyard, Vogel, & Luck, 1998). This theory suggests that signal enhancement (i.e., facilitation) works as a gain control, increasing the sensitivity of neurons to properties of the attended stimulus, relatively enhancing processing of stimuli at attended locations as compared to unattended locations (Handy & Khoe, 2005; Hillyard et al., 1998; Mangun & Hillyard, 1991; Mangun, 1995; Navalpakkam & Itti, 2007; Russo et al., 2003). However, recent research suggests that suppressive mechanisms may also contribute to visual selective attention (Couperus & Mangun, 2010). Suppression may contribute to selection by reducing the sensitivity of neurons to the properties or spatial location of the unattended stimulus during stimulus processing similar to facilitation. Both facilitation and suppression are supported by processes both prior to stimulus onset (preparatory facilitation/suppression) as well as during stimulus processing (Couperus & Mangun, 2010). Visual search, like selective attention in general, often utilizes top-down control to direct attention to the relevant stimuli while simultaneously suppressing processing of irrelevant stimuli.

In the context of visual search, selective attention has often been examined through the N2 posterior contralateral component (N2pc). The N2pc is recorded over lateral occipital scalp regions when a search display appears and is thought to reflect selection of the attended item in the context of distractors. Moreover, it is thought to reflect voluntary selective attention that is guided by top-down processes (Eimer, Kiss, & Nicholas, 2011). The component is seen approximately 175–300 ms after the onset of the display and selection of the attended item is reflected in greater negative activity contralateral as compared to ipsilateral to the attended item. While the majority of studies suggest this component reflects attentional-filtering operations (Boehler, Tsotsos, Schoenfeld, Heinze, & Hopf, 2011; Eimer, 1996; Hopf, Boelmans, Schoenfeld, Heinze, & Luck, 2002; Luck & Hillyard, 1994a, b; Luck, Girelli, McDermott, & Ford, 1997), there remain questions regarding the relative contributions of selective enhancement and suppression to this process (Eimer, 1996; Hickey, Di Lollo, & McDonald, 2009).

In contrast to research on adults, far less is known about the neurological correlates of visual selective attention in children, particularly in relation to visual search and top-down attentional control processes required for visual search (Booth et al., 2005; Taylor & Khan, 2000). One aspect that has been explored is early attention effects at the P1 visual component. For example, Taylor and Khan (Taylor & Khan, 2000) compared parallel and serial visual search in children aged 7–12 years. Shorter latencies were found for the P1 during pop-out searches when a target was present in the display compared with when it was not, suggesting latency attention effects during the visual search as early as the P1 in children. In other non-search-selective attention tasks, the P1 visual component has shown robust amplitude modulation by attention in children across a wide range of ages (Couperus, 2011; Harter, & Anllo-Vento, 1991; Rueda et al., 2004). However, unlike non-search studies, visual search studies in children have not found amplitude changes in the P1 in relation to attention (e.g., Taylor & Khan, 2000). Moreover, Taylor and Khan (2000) did not examine the N2pc, nor has any other study of visual search in children. As this component indexes selective attention in the presence of distractors and requires voluntary top-down controlled attentional processes, it is an ideal candidate to better understand selective attention during visual search in children.

Thus, in this study children completed a visual search task that includes distractors to examine voluntary visual selective attention through activity at the N2pc. Specifically, the task used requires participants to use a top-down attention set to guide attention, based on color, to a target among distractors. As the N2pc in children has not yet been studied it may be possible that we will not see an N2pc at all. However, based on behavioral studies of visual search we hypothesize that children will show an N2pc, but may show amplitude differences in the N2pc due to differences in selective attention mechanisms that are reflected in this component. We also anticipate greater overall amplitude and delayed onset of the N2pc in children due to increases in myelination across development (Casey, Tottenham, Liston, & Durston, 2005; Turken et al., 2008).

## Methods

### Participants

Twenty-one adults (mean age = 19.52 years, SD = 1.25, 14F/7M, 16 White, two White/Asian, one Asian/Pacific Islander, one White/Hispanic, and one Hispanic) and nineteen 9- to 12-year-old children (mean age = 10.52 years, SD = 1.07, 5F/14M, 16 White, one White/African American, one Asian, and one Hispanic) participated in this study. Participants were recruited from the Pioneer Valley in Western Massachusetts. Participants were excluded from participation if they had visual impairments that could not be corrected with glasses/contacts, if they were born premature (i.e., less than 36 weeks), had or were suspected of having a learning disability, had or were suspected of having a clinical mental health diagnosis, or were on psychotropic medications as indicated by either self or parent report. All participants also reported being right-handed. All adult participants gave written consent prior to participation. Child participants were given a verbal explanation of all aspects of informed consent and then gave written assent to participate. Additionally, parents provided written consent for their children to participate. All consents and research procedures were approved by the Hampshire College Institutional Review Board. All participants were compensated with $10–20 for their time.

### Visual search task

**Fig. 1:**
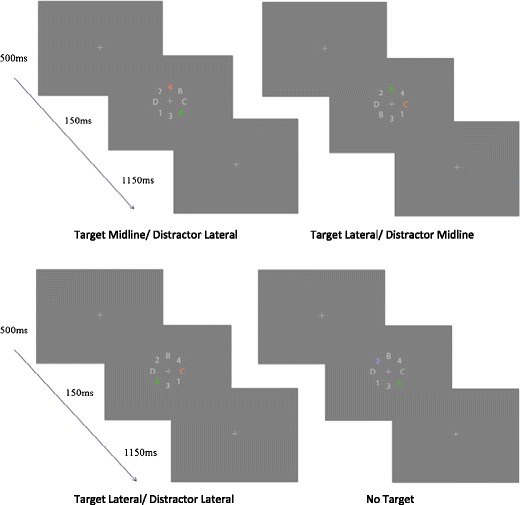
Stimulus display for the task when the target color is red

The visual search task was based on the task used in a study by Eimer, Kiss, and Nicholas (2011). This specific task was chosen to parallel previous work with the N2pc in adults and to ensure data could be compared across studies to validate findings. Following verbal and visual instructions participants were asked to complete 768 trials presented in eight blocks of 96 trials with breaks provided between blocks. There were no practice blocks. Break length was determined by the participant, typically lasting 30 s to 1 min for adults and slightly longer for children. During each trial they were presented with a fixation of 500 ms followed by a search display for 150 ms consisting of eight items (letters and numbers) surrounding the fixation cross. Participants were then shown the fixation for another 1,150 ms during which they were asked to indicate if an attended item (if present) was a letter or number.^1^ The eight search items presented were the numbers 1, 2, 3, and 4 and letters A, B, C, and D, and were created in Photoshop CS5. Each search display contained six of these numbers/letters in gray (RGB = 188, 188, 188, L:76) while the remaining two were presented in two of three possible colors, red (RGB = 225, 145, 115, L:68), green (RGB = 81, 188, 37, L:68), or blue (RGB = 171,159,213, L:68) (see Fig. 1). All stimuli, including the fixation cross, were .573 × .573 degrees visual angle in size. The numbers and letters appeared at an eccentricity of 2.39° visual angle from the fixation cross. There were 256 displays of each color combination (red/green, red/blue, green/blue). Participants were asked to attend to only one of the three colors for the duration of the task (counterbalanced across participants). Additionally, they were asked to press either the left or right mouse button using either one or both hands (when using both hands the thumbs were used) to indicate if the attended item was a letter or a number. If no items were in the attended color they were asked to not respond and wait until the next display. Thus, approximately two-thirds of the displays contained a target-attended item while the remaining one-third did not (numbers are approximate as trials were chosen randomly from all possible displays). The location of colored items varied pseudo-randomly to create seven possible conditions as a function of the color attended: Target midline/Distractor left, Target midline/Distractor right, Distractor midline/Target left, Distractor midline/Target right, Target left/Distractor right, Target right/Distractor left, and No target present. The target present conditions were then collapsed for analysis to create three possible conditions: Target lateral/Distractor lateral (TLDL), Target lateral/Distractor midline (TLDM), and Target midline/Distractor lateral (TMDL).

### Electrophysiological methods

Scalp electroencephalograms (EEGs) were recorded using tin electrodes embedded in an elastic cap (Electro-cap International). The 32 electrodes were located at standard sites of the International 10–20 system of electrode placement (Jurcak, Tsuzuki, & Dan, 2007) as follows: FPZ, FZ, CZ, CPZ, PZ, OZ, FP1, FP2, F7, F8, F3, F4, FT7, FT8, T7, T8, C3, C4, TP7, TP8, CP3, CP4, P7, P8, P3, P4, O1, O2, HEOG, VEOG. Electroencephalograms were recorded and referenced to the right mastoid and impedances were kept below 5 k ohms for all participants. The mastoid reference is preferred when using a smaller number of channels because an average reference (mean of recorded electrodes) is not as accurate under such conditions (Handy, 2005). The EEGs were amplified using a Synamps2 Amplifier with a bandpass filter of 0.1 to 100 Hz, and digitized at a sampling rate of 500 samples/s. To ensure eye fixation, electro-oculograms (EOG) were recorded for both vertical and horizontal eye movements (electrodes were placed inferior to the left eye and both to the left and right of the outer canthus).

### Data analysis and reduction

Behavioral data were collected for both accuracy and reaction time. Accuracy and reaction time data is based on target present displays.

EEGs were re-referenced off-line to linked mastoids, sorted into epochs (200 ms pre-stimulus to 1,000 ms post-stimulus), and artifact-free trials were averaged to yield event-related potentials (ERPs) for the various conditions where a target was present (TLDL, TLDM, and TMDL). Only trials with a correct response were used; error trials were excluded from ERP analyses. The ERPs were baseline-corrected using the mean of the 200-ms pre-stimulus period. Artifact rejection involved the automated exclusion of trials if they contained significant ocular artifacts, muscle, or movement artifacts as reflected by amplitudes ± 50 μvolts for adults and ± 100 μvolts for children at FP1, FP2, vertical, or horizontal eye electrodes. Additionally, trials were rejected if activity was greater than ± 100 μvolts for adults and 150 μvolts for children at all other electrodes. Channels that were consistently bad across the experiment as determined by visual inspection were marked as such and not used in analyses. Participants were eliminated from analyses if they did not complete the full 768 trials, 10 % or more channels were bad (Picton et al., 2000), if residual eye artifacts exceeded 5 μvolts, or had less than 25 artifact free trials in more than half of the analyzed conditions. Out of an initial 26 adults and 24 children recruited, five adults and five children were excluded based on these criteria, resulting in 21 adults and 19 children included in analyses (average number of trials adults = 52.92, SD = 23.2, children = 53.94, SD = 24.0, this corresponds to approximately a 37 % rejection rate). One additional participant was left out of behavioral analysis as the individual reversed the response buttons.

The N2pc was defined based on both the grand averages of each age group as well as the contralateral-ipsilateral difference waveforms. For adults the N2pc was seen in the window 220 to 270 ms and for children the N2pc was defined by the window 240 to 290 ms. Mean amplitude and latency data were collected for all conditions. Latency was determined in adults by the latency to peak in the entire N2pc window. In contrast, latency in children was determined by visual determination of the latency to peak of the peak closest to the middle of the window (i.e., 265 ms) as a rise to a secondary peak following the N2pc would otherwise skew the data. Adult data was visually inspected to ensure similar skew by neighboring peaks was not an issue. Repeated-measures ANOVAs were used for both mean amplitude and latency to examine the N2pc as a function of target and distractor location as well as a function of age.

## Results

### Behavioral analyses

Reaction time and accuracy data were examined to ensure participants were completing the task similarly and accurately. A 2 (group: adult vs. child) × 3 (target/distractor location) repeated-measures ANOVA was used in analyses. Accuracy data showed no significant effects of condition or interactions, but did show a significant effect of age with mean accuracy for adults higher than for children (F(1,37) = 12.48, p =.001, η_p_^2^ = .252; adults: TMDL = 82.8, SD = 5.9, TLDM = 82.8, SD = 5.8, TLDL = 82.4 SD = 4.5; children: TMDL = 74.0, SD = 10.1, TLDM = 73.5, SD = 12.0, TLDL = 73.42, SD = 10.5). Reaction time data also showed a significant main effect of age (F(1,37) = 7.52, p =.009, η_p_^2^ = .169) with no other main effects or interactions. This age effect reflects improvements in reaction times with age (adults: TMDL = 513.28, SD = 103.2, TLDM = 517.24, SD = 96.7, TLDL = 525.36 SD = 90.1; children: TMDL = 610.53, SD = 121.0, TLDM = 612.36, SD = 124.3, TLDL = 612.31, SD = 115.33).

### N2 posterior contralateral component (N2pc) amplitude

**Fig. 2:**
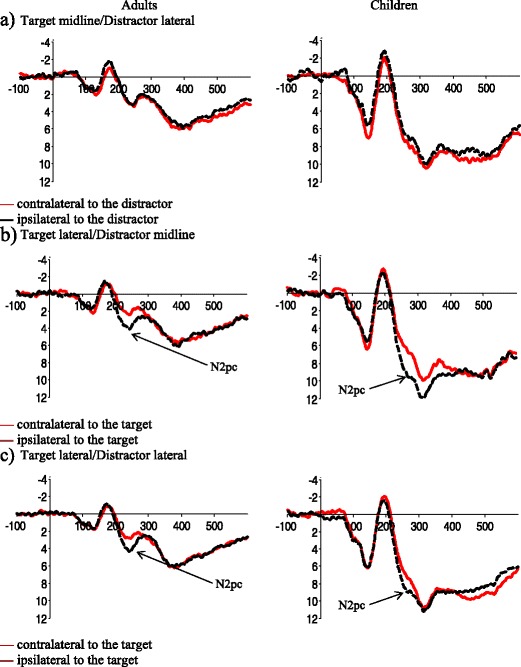
Event-related potentials (ERPs) elicited in the 600-ms interval after search array onset at posterior electrode sites P7/8 in adults (left column) and children (right column). (**a**) ERPs for search arrays containing a midline target and lateral distractor, (**b**) lateral target and midline distractor, and (**c**) lateral target and lateral distractor

To examine the N2pc, a 2(age) × 3(target/distractor location) × 2 (hemisphere: contralateral or ipsilateral to the target^2^) repeated-measures ANOVA was performed for the electrode pair P7/P8. There was a significant main effect of age (P7/P8, F(1,38) = 4.81, p = .035, η_p_^2^ = .112). Additionally, there was a significant interaction between the target/distractor location and the hemisphere of processing (i.e., contralateral or ipsilateral) (P7/P8, F(1,76) = 17.43, p < .001, η_p_^2^ = .314) as well as a three-way interaction between age, target/distractor location, and hemisphere of processing (P7/P8, F(1,76) = 5.24, p = .011, η_p_^2^ = .121). To better understand the interactions at P7/P8, post-hoc analyses were performed within each age as there are large differences in overall activity between these two groups (see Fig. 2).

### Adults

A 3(target/distractor location) × 2(hemisphere: contralateral or ipsilateral to the target) repeated-measures ANOVA showed a significant main effect of target/distractor location (F(2,40) = 3.84, p = .039, η_p_^2^ = .161) as well as an interaction between the target/distractor location and the hemisphere of processing (P7/P8, F(2,40) = 4.19, p =.032, η_p_^2^ = .173) similar to previous studies (Eimer et al., 2011). Additional follow-up repeated-measures ANOVA for each of the target/distractor locations suggests this interaction results from the presence of the N2pc when both the target and distractor were lateral (TLDL, F(1,20) = 4.34, p =.05, η_p_^2^ = .178) as well as when only the target was in a lateral position (TLDM, F(1,20) = 5.54, p =.029, η_p_^2^ = .217) while there was no N2pc when the target was in the midline position (TMDL, p > .05).

### Children

A 3(target/distractor location) × 2(hemisphere: contralateral or ipsilateral to the target) repeated-measures ANOVA showed a significant interaction between the target/distractor location and the hemisphere of processing (P7/P8, F(2,36) = 12.57, p < .001, η_p_^2^ = .411). Additional follow-up repeated-measures ANOVAs for each of the target/distractor locations suggests this interaction results from the presence of the N2pc when both the target and distractor were lateral (TLDL, F(1,18) = 11.32, p =.003, η_p_^2^ = .386) as well as when only the target was in a lateral position (TLDM, F(1,18) = 10.26, p =.005, η_p_^2^ = .363), while there was no N2pc when the target was in the midline position (TMDL, p > .05) similar to adults.

### N2 posterior contralateral component (N2pc) latency

To examine the latency of the N2pc, a 2(age) × 3(target/distractor location) × 2(hemisphere: contralateral or ipsilateral to the target) repeated-measures ANOVA was performed for the electrode pair P7/P8. There was a significant main effect of age (P7/P8, F(1,38) = 144.5, p < .001, η_p_^2^ = .792, adults = 242.5, SD = 10.6, children = 263.7, SD = 5.6). As latency data of this type is inherently noisy, results were confirmed using a jack-knife procedure (F_c_(1,38) = 37.17, p < .001) (Ulrich & Miller, 2001).^3^ There were no other significant main effects or interactions.

## Discussion

While previous research has suggested even young children utilize processes similar to adults during visual spatial attention (Couperus, 2011), there is limited knowledge concerning the processes of selective attention underlying visual search in children (Taylor & Khan, 2000). Thus, this study focused on the N2pc, a component typically associated with voluntary top-down guided selective attention during visual search tasks (Eimer, 1996; Hickey et al., 2009; Jannati, Gaspar, & McDonald, 2013; Luck & Hillyard, 1994a, b). Findings of this study replicate previous findings with adults demonstrating an N2pc when targets were in lateral positions and a distractor was present as compared to when the target was in a midline position (Eimer et al., 2011). Additionally, while age did affect overall latency and amplitude as well as subtly moderate attention affects as a function of target location, children showed similar patterns of activity in relation to when an N2pc was or was not present. The presence of the N2pc in children suggests that the processes associated with the N2pc are functional in children aged 9–12 years. Specifically, the presence of an N2pc to lateral targets implies effective top-down control and selection and the absence of the N2pc to the lateral distractors suggests good attentional control during visual search which has not previously been shown in children.

**Fig. 3:**
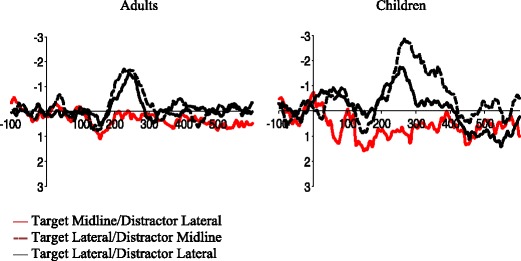
Difference waves at posterior electrode sites P7/8 in adults (left column) and children (right column) in the 600-ms interval after search array onset

Children in this study showed both greater overall activity and delayed onset of the N2pc. These differences are similar in magnitude to previous studies (Couperus, 2011) and are thought to reflect myelination across childhood that increase both efficiency and speed of processing (Casey et al., 2005; Turken et al., 2008). However, interestingly, while both children and adults showed an N2pc to lateral targets and no N2pc to lateral distracters, there was an additional main effect of target location on amplitude for adults which may suggest a stronger distinction between location conditions in adults as compared to children. There are several possible explanations this difference between adults and children. First, children’s data is more variable than adults’ from trial to trial, potentially weakening effects. Second, as can be seen in the difference waves (Fig. 3), while the difference between contralateral and ipsilateral activity was similar in the two target lateral conditions in adults, for children there was greater variability with the target lateral/distractor midline condition producing a stronger difference than the target lateral/distractor lateral condition. This difference may suggest that children process the two target lateral conditions differently despite both showing an N2pc. While it is not clear why children may show stronger activity when the distractor is midline as compared to lateral, one possibility is that despite good attentional control the salient distractor does capture some attention as it is the same salience as the target, producing an inverted N2pc that in turn reduces the target N2pc when it is in the lateral position. One way to examine this possibility in future studies is to reduce the saliency of the distractor.

Additionally, while adults and children show the same overall pattern of processing during early perceptual stages of visual search, this research should only be taken as a first step in understanding the underlying processes of visual search in children. In the adult literature there is currently debate concerning the N2pc and the processes that underlie the component. While some argue the component reflects selective enhancement of the attended items (Eimer, 1996), others argue that it reflects a combination of both selective enhancement of the attended items as well as suppression of the unattended items (Hickey, Di Lollo, & McDonald, 2009; Jannati, Gaspar, & McDonald, 2013). While this study was not designed to decompose the N2pc into separate processes, some studies have attempted to do so. For example, Hickey et al.’s (2009) research suggests the N2pc reflects both a positive distractor suppression component, or distractor positivity (P_D_), and a target negativity (N_T_). While Hickey et al. propose that the P_D_ reflects suppressive processes, it is not clear if the (N_T_) reflects enhancement or suppressive activity related to the target stimulus. Future research might focus on such components to see if differences between adults and children in effect sizes are a result of differences in the underlying components of the N2pc. Additionally, while as noted above, research suggests overall amplitude and latency differences are likely a function of myelination changes across development, it is possible that additional differences are masked by these overall changes and may be uncovered with a finer-grained exploration of the N2pc in children.

Moreover, while not a focus of this study, there were several additional aspects to the data that are important to note. First, while no amplitude effects at P1 were hypothesized given that effects had not been shown in previous visual search studies (Taylor & Khan, 2000), both adults and children showed main effects of attention (F(1,38) = 9.10, p = .005., η_p_^2^ = .193) as well as target/distractor location (F(2, 76) = 4.72, p = .013, η_p_^2^ = .110). This may be due to a small sensory imbalance on trials where only the target or distractor was lateral as described by Hickey, McDonald, and Theeuwes (2006). Evidence for this comes from follow-up comparisons that show the attention effect was primarily in the target midline/distractor lateral condition (Adults t(20) = 3.21, p = .004, Children t(18) = 2.70, p = .015). However, as effects at the N2pc were seen in both target lateral/distractor midline conditions and target lateral/distractor lateral conditions it cannot be argued that any potential sensory imbalances were responsible for the N2pc findings. Second, visual inspection of the data suggests the existence of a component just beyond the N2pc that also may show effects of condition. This additional component is seen in children’s data, but is not seen in adult data. Both of these additional effects should be investigated further in future studies as they may suggest differences between children and adults in both earlier and later processing during visual search.

Finally, it is possible that while processes that underlie visual search appear to be functional in the task presented in this study, the conditions under which it is functional may be different in children as compared to adults. The task used in this study required the filtering of only one distracting unattended item of a salient nature (i.e., in a color of equal luminance to the target) and six other non-task-related items. It is possible that with greater numbers of unattended but salient distractors, increases in the complexity and/or salience of the attended and unattended stimuli, or other manipulations of target and distractors, differences may yet be seen between adults and children. Additionally, Donnelly et al. (2007) showed differences in visual search in slightly younger children than were used here, showing differences only in 6- to 7-year-old children. Thus it is possible that younger children may still show differences in the N2pc that are not seen in older children. However, while future studies should further examine each of these possibilities, results of this study demonstrate that children between 9 and 12 years of age utilize top-down attentional sets to guide attention as a function of color and similar mechanisms to adults in support of visual selective attention during visual search as indexed by the N2pc. Thus the N2pc can be used as a marker of attentional selection in children.

## Footnotes

1 Perceptually, aside from the first and last trials as there was no break between trials, participants experienced the trials as 1,650 ms of fixation between each display.

2 For the midline target condition ipsilateral and contralateral are defined in relation to the distractor instead of in relation to the target.

3 Latencies used for the jack-knife procedure were determined by visual inspection. Each condition for each participant was examined during the 240- to 290-ms window and the latency was determined for the maximum value of the peak identified as the N2pc.
